# Boxing behavior recognition based on artificial intelligence convolutional neural network with sports psychology assistant

**DOI:** 10.1038/s41598-024-58518-5

**Published:** 2024-04-01

**Authors:** Yuanhui Kong, Zhiyuan Duan

**Affiliations:** School of Science of Physical Culture and Sports, Kunsan University, Kunsan, 54150 Korea

**Keywords:** Sports psychology, Convolutional neural network, Artificial intelligence, Boxing behaviour, Action recognition, Computational science, Computer science, Information technology, Scientific data

## Abstract

The purpose of this study is to deeply understand the psychological state of boxers before the competition, and explore an efficient boxing action classification and recognition model supported by artificial intelligence (AI) technology through these psychological characteristics. Firstly, this study systematically measures the key psychological dimensions of boxers, such as anxiety level, self-confidence, team identity, and opponent attitude, through psychological scale survey to obtain detailed psychological data. Then, based on these data, this study innovatively constructs a boxing action classification and recognition model based on BERT fusion 3D-ResNet, which not only comprehensively considers psychological information, but also carefully considers action characteristics to improve the classification accuracy of boxing actions. The performance evaluation shows that the model proposed in this study is significantly superior to the traditional model in terms of loss value, accuracy and F1 value, and the accuracy reaches 96.86%. Therefore, through the comprehensive application of psychology and deep learning, this study successfully constructs a boxing action classification and recognition model that can fully understand the psychological state of boxers, which provides strong support for the psychological training and action classification of boxers.

## Introduction

### Research background and motivations

As a highly technical and psychological sport, boxing has always attracted extensive attention. In the past decades, sports psychology has made remarkable progress in analyzing athletes' behavior and improving their performance^1,2^. With the rapid development of artificial intelligence (AI) technology, especially the application of convolutional neural network (CNN), researchers began to introduce these technologies into the field of sports behavior recognition^3–5^. Therefore, paying attention to the behavior recognition of boxing and exploring the influence of psychological characteristics of boxers on behavior recognition have become the focus of scholars in related fields.

The motivation of the study is to deeply understand the psychological state and behavior characteristics of boxers in the competition, which is helpful to improve the accuracy and individualization level of boxing training^6–8^. By integrating AI technology into the boxing behavior recognition model, it is expected to capture and understand the complex behaviors of athletes in the boxing process more comprehensively^9,10^.

## Research objectives

The goal of this study is to establish a boxing behavior recognition model based on AI-CNN, and on this basis, combined with sports psychology factors, explore the influence of athletes' psychological state on the accuracy and efficiency of behavior recognition. The objectives and innovations of this study are mainly reflected in the following aspects:

1. In-depth study on the psychological characteristics of boxing: This study not only pays attention to the physical movements of athletes, but also deeply studies the psychological characteristics that affect boxing performance, such as anxiety and concentration, and develops the corresponding sports psychological measurement tools. This provides a new perspective, that is, the psychological state of athletes is directly related to their performance, which provides a new path for follow-up research.
2. By using advanced video capture technology to obtain the behavior data of boxers, this study provides a rich sample for the training of AI model. These high-quality data samples are the basis of model recognition and analysis of boxing movements, ensuring the accuracy and efficiency of recognition results.
3. By combining the psychological statistical text processed by BERT algorithm and the video image features extracted by 3D-RESNET network, the model of this study can not only understand the athletes' actions from the technical level, but also deeply analyze their psychological state, thus achieving a new breakthrough in boxing behavior recognition. This multi-modal fusion method provides a new perspective for boxing action classification and athlete state analysis, and expands the application scope of AI in the field of sports behavior analysis.

Therefore, this study constructs a comprehensive research framework, which combines sports psychology with AI to improve the in-depth understanding and recognition level of boxing behavior. This plays a positive role in the future boxing training, sports psychology research and the development of AI in the field of sports behavior analysis.

## Literature review

The history of sports psychology in boxing research originated from the in-depth study of athletes' behavior and psychological state. Many scholars have studied the importance of anxiety level, self-confidence and concentration in boxing performance. Sachkova and Volkov^11^ discussed the influence of group identity on the sports achievement level of young boxers, and emphasized the importance of psychological factors in boxing performance. Prabowo et al.^12^ verified the effectiveness of sports anxiety test in amateur boxers. Korobeynikov et al.^13^ paid attention to the influence of cognitive function and special work ability on elite boxers, and emphasized the importance of cognitive ability on boxing performance. Kovalev et al.^14^ thought that psychological gender has a significant impact on the competitive performance of female boxers. Osipov et al.^15^ emphasized the importance of psychological factors in women's boxing, which has practical implications for sports psychology and gender research. Sato et al.^16^ discussed the experience of kendo athletes in fencing, highlighting the athletes' experience in kendo sports. Schinke et al.^17^ emphasized the importance of scientific practice in the field of sports psychology, which provides guidance and direction for sports psychologists to better support athletes.

The application of AI technology in the field of sports behavior recognition has attracted much attention in recent years. The development of machine learning and deep learning algorithms provides new possibilities for automatic recognition of action behavior. Verma et al.^18^ summarized the application of supervised and unsupervised machine learning in suspicious behavior identification in intelligent monitoring system. Kulsoom et al.^19^ highlighted the flexibility of machine learning in different application fields and provides a comprehensive analysis for sports behavior recognition. Zhao et al.^20^ emphasized the effectiveness of deep learning in improving the accuracy and sensitivity of sports behavior recognition. Xin^21^ highlighted the application prospect of new technology in sports analysis and provides a new idea for improving the automatic recognition of basketball sports behavior. Coelho et al.^22^ emphasized the application of machine learning in materials science and engineering, which provided feasibility for real-time monitoring of sports behavior. Wang et al.^23^ emphasized the prospect of combining optical sensing technology with machine learning in sports behavior monitoring. Yang et al.^24^ introduced the flexible strain sensor used in handwriting recognition based on machine learning, which highlighted the application potential of machine learning in realizing high-precision handwriting recognition. Zhang et al.^25^ proposed a lower limb action recognition method based on Vision Transformer, which highlighted the innovation of deep learning algorithm in action behavior recognition based on sensor data.

Convolutional neural network (CNN), as an important branch of deep learning, has shown many advantages in sports behavior analysis. Its ability to process images and time series data makes it outstanding in video analysis and action trajectory recognition. Andrade-Ambriz et al.^26^ thought that time series CNN has achieved good performance in action behavior analysis, which provided empirical support for the application of deep learning in action recognition. Arab et al.^27^ used millimeter-wave Doppler radar and CNN to realize human action recognition and classification. Muaaz et al.^28^ used CNN to extract information from Wi-Fi signals, which provided an intelligent sports behavior monitoring method for health information systems without wearing equipment. Gholamiangonabadi and Grolinger^29^ established a personalized wearable sensor human activity recognition model, which highlighted the effectiveness of convolutional neural network in customized sports behavior analysis. Gangrade and Bharti^30^ realized a vision-based Indian sign language gesture recognition by using CNN. Pathan et al.^31^ used multi-head CNN to fuse images and hand marks and sign language recognition. Zhang et al.^32^ used deep learning and transfer learning to realize online electromyography gesture recognition. CNN showed superior performance in cross-session gesture recognition and provided an innovative solution for real-time gesture control. Jin et al.^33^ proposed a novel ECG signal denoising method based on deep wavelet CNN, and emphasized the application of CNN in biomedical signal processing.

To sum up, through the research and analysis of the above scholars, this study discusses the importance of psychological factors such as group identity and gender differences to boxers, and introduces the application of AI technology in the field of sports behavior recognition. Finally, it summarizes the advantages and limitations of convolutional neural network in sports behavior analysis. However, the research on the combination of sports psychology and AI applied to behavior recognition is relatively rare. Therefore, while integrating sports psychology and AI technology, this study pays attention to the improvement of deep learning algorithm, which provides a comprehensive and innovative perspective for the research in the field of boxing behavior recognition.

## Research model

### Psychological characteristics and data collection and analysis in boxing

**Figure 1:**
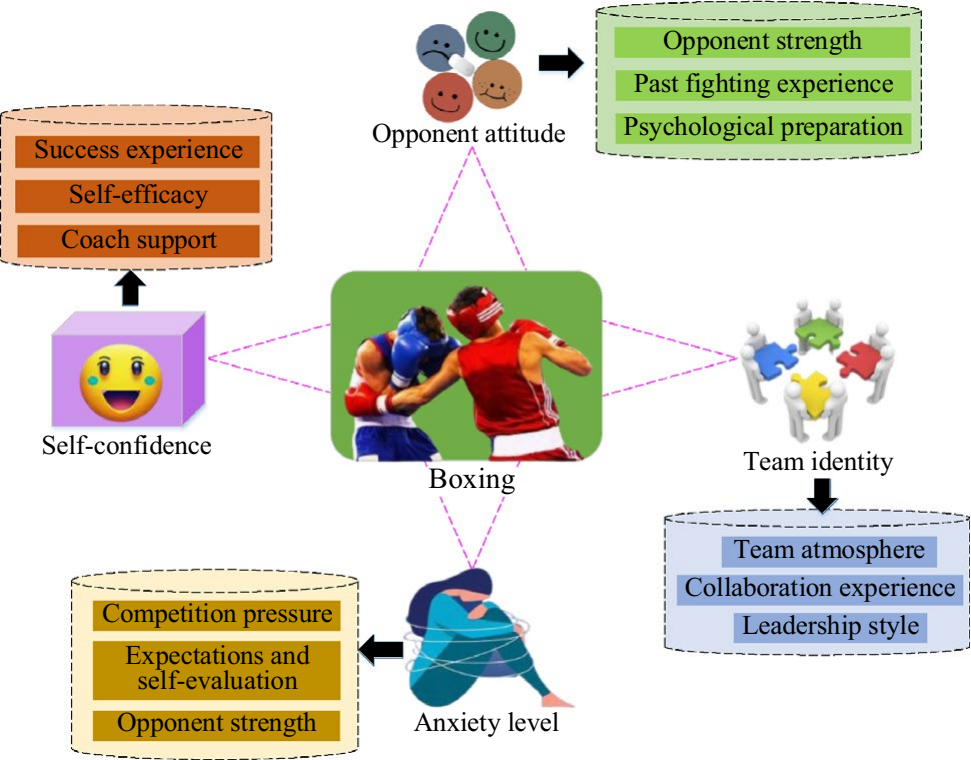
Schematic diagram of psychological characteristics and influencing factors of boxers.

Boxing is a high-intensity sport, which requires athletes to keep calm, concentrate, deal with pressure and maintain self-confidence in the competition^34,35^. Factors such as anxiety level, self-confidence, team identity and opponent's attitude may have a significant impact on the performance of boxers. Understanding and quantifying these psychological characteristics is very important for personalized training and improving athletes' competitive level^36–39^. The psychological characteristics and influencing factors of boxing are shown in Fig. 1.

In this study, in order to effectively understand the psychological state of boxers, reference and research, when designing the psychological scale of boxing, it is needed to consider the dimensions and specific manifestations of various psychological characteristics. Table 1 shows the design of psychological scale, covering four dimensions: anxiety level, self-confidence, team identity and opponent's attitude.

**Table 1:** Boxer psychological scale.

	Question	Very agree	Agree	General	Disagree	Very disagree
Anxiety level	A1: I feel anxious the day before the game					
	A2: I feel anxious when I enter the competition field					
	A3: I feel anxious when facing a strong opponent					
Self-confidence	B1: I believe I have enough skills and strength					
	B2: At the critical moment, I can remain calm and confident					
	B3: I have positive expectations for my performance					
Team identity	C1: I think my team is a close cooperative group					
	C2: In difficult times, I feel that my team supports me					
	C3: I am proud to be in this team					
Opponent attitude	D1: I have confidence in facing a strong opponent					
	D2: I think the improvement of my opponent's strength is an opportunity for my own progress					
	D3: I can keep calm and rational during the competition					

In this questionnaire, the respondents are boxers, and the investigation time is from March 1, 2023 to June 1, 2023. The total number of questionnaires distributed was 452, of which 431 questionnaires were recovered, with a recovery rate of 95.35%, which indicated that this questionnaire was an effective survey. Results Likert five-level scale method was used, and the answer options were divided into five types: very agree = 5, agree = 4, general = 3, disagree = 2, and very disagree = 1. The whole questionnaire survey process strictly follows ethical principles, and does not involve personal privacy. All participants are over 18 years old, and the participation process is carried out with the consent of the participants. And use SPSS 24.0 software for statistical analysis.

### Analysis of boxing action classification and recognition model based on CNN assisted by sports psychology

In this study, an innovative boxing action classification and recognition model is proposed, aiming at combining sports psychology with advanced deep learning technology. In this study, an innovative model is proposed, which combines the BERT algorithm to process text data and the 3D-RESNET network to extract the spatial–temporal features of video images, aiming at a more comprehensive understanding of athletes' psychology and action state. The innovation of this method is that it provides a brand-new research perspective for boxing action classification by fusing psychological statistical text and video image data.

Firstly, the BERT algorithm^40,41^ is adopted for athletes' psychological statistical texts, which is a pre-trained deep bidirectional transformer model, specially designed to understand the complex context of natural language texts. By pre-training large-scale text data, BERT model can capture rich language features and provide strong support for understanding athletes' psychological state. In order to further improve the model's ability to deal with boxing-specific texts, a self-defined attention mechanism layer is added on the basis of BERT, so that the model can pay more attention to the text information closely related to boxing behavior, and thus capture the psychological characteristics of athletes more accurately.

Secondly, for the processing of boxing video images, 3D-RESNET network^42–44^ is selected. Compared with the traditional 2D convolutional network, 3D-RESNET can effectively extract the spatial–temporal features in video sequences by introducing the time dimension, which is very important for understanding continuous actions and behavior patterns. The original architecture of 3D-RESNET is optimized, including adding a deeply separable convolution layer to reduce the complexity of the model, and introducing a time attention module to improve the ability to capture the time dependence of action sequences. These improvements enable the network not only to process video data more efficiently, but also to identify and classify complex boxing movements more accurately.

By fusing the text data processed by BERT algorithm with the video image features extracted by 3D-RESNET network, the new model can fully understand the athletes' state from two dimensions: on the one hand, it can analyze the athletes' psychological state and strategy choice from psychological texts. On the other hand, it captures athletes' action details and technical execution through video images. This multi-modal fusion method provides a new perspective for boxing action classification and athlete state analysis, and is expected to open new research directions in sports science and human–computer interaction.

**Figure 2:**
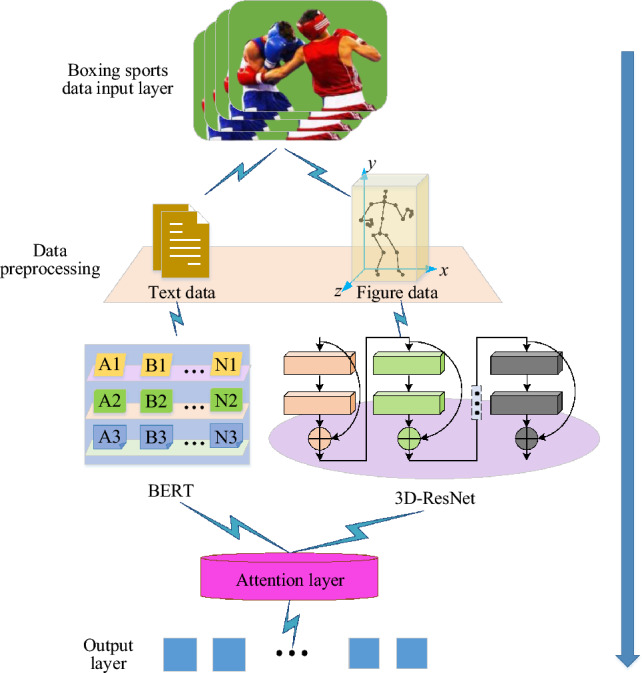
Schematic diagram of boxing action classification and recognition model based on BERT fusion 3D-ResNet.

Among them, the boxing action classification and recognition model based on BERT fusion 3D-ResNet is shown in Fig. 2.

In Fig. 2, in this model, BERT algorithm is used to input the obtained psychological statistical text of boxers into the model according to the psychological scale and convert it into a real-value vector representation. In this study, an attention layer based on self-attention mechanism is introduced, aiming at effectively integrating the feature representations from BERT (processing text data) and ResNet-3D (processing video sequence data), and calculating the attention weight at each time step through a series of neural network layers. The design of this layer is inspired by the Transformer architecture, which dynamically adjusts the importance of feature representation by calculating the attention scores between different input elements.

In practice, firstly, text and video features are extracted from BERT and ResNet-3D models, respectively. For BERT, the output of the last layer is obtained as the text feature vector. For ResNet-3D, the output of the last layer of the network is also extracted as the video feature vector. Then, the two feature vectors are connected to form a unified feature representation. On this basis, the "attention layer" is introduced to act on this unified feature representation. Firstly, it calculates the importance of each element in the feature for boxing behavior recognition, and this process is realized by training the weight matrix. Then, according to the calculated attention score, the feature vectors are weighted and summed to generate an attention-weighted feature representation. This weighted feature representation captures the most critical information in text and video data, and provides richer and more accurate feature input for subsequent classification tasks. This attention mechanism enables the model to capture the key information related to boxing action in video sequences more effectively, thus improving the performance and generalization ability of the model.

Assuming that the one-hot vector corresponding to the input sequence *x* is represented as $e^{t} \in R^{N \times \left| V \right|}$, its word vector $E^{t}$ can be represented by Eq. (1):

1 $$E^{t} = e^{t} W^{t}$$

$W^{t} \in R^{\left| V \right| \times e}$ refers to the trained word vector matrix. $\left| V \right|$ refers to the vocabulary size, and *e* refers to the quantity dimension.

The block vector is usually used to record which partition the word to be encoded belongs to. The block vector $E^{s}$ is expressed as Eq. (2):

2 $$E^{s} = e^{s} W^{s}$$

$E^{s}$ refers to converting block coding into real-valued vectors through block vector matrix $W^{s}$. $W^{s} \in R^{\left| S \right| \times e}$ refers to block vector matrix, and $\left| S \right|$ refers to the number of blocks.

The position vector is used to encode the absolute position of each word, which can be expressed as Eq. (3):

3 $$E^{p} = e^{p} W^{p}$$

$W^{p} \in R^{N \times e}$ refers to the position vector and *N* refers to the maximum position length.

In this model, 3D-ResNet network is used to classify boxing movements by identifying video images. ResNet-50 is used as the backbone network. ResNet-50 includes an initial convolution layer, followed by 16 residual blocks, each of which consists of 3 convolution layers, with a total of 48 convolution layers, plus an initial convolution layer, and an average pooling layer, the total number of layers reaches 50. This depth enables the network to capture rich feature representations, and the design of its residual connection is helpful to alleviate the problem of gradient disappearance in deep network training.

For 3D-CNN, a network structure consisting of four convolution layers is designed, and each convolution layer is followed by Batch Normalization and ReLU activation function. After every two convolution layers, a 3D maximum pooling layer is used to reduce the feature dimension and extract more abstract features. Finally, the final action category prediction is output through a fully connected layer. $Q = \left( {q_{1} ,q_{2} , \cdots ,q_{T} } \right) = \left\{ {q_{i} } \right\}_{i = 1}^{T}$ is used to refer to boxing sports video with *T* frames, and sliding window is used to divide it into a sequence $V^{N}$ of *N* boxing video segments, as shown in Eq. (4):

4 $$V^{N} = \left( {v_{1} ,v_{2} , \cdots ,v_{N} } \right)$$

Each video segment $v_{i}$ is input into 3D-ResNet to extract its corresponding fixed-length video feature expression $f_{i} \in R^{d}$, and the input sequence can be expressed as Eq. (5):

5 $$F^{N} = \left( {f_{1} ,f_{2} , \cdots ,f_{N} } \right) = \left\{ {\Phi_{\theta } \left( {v_{i} } \right)} \right\}_{i = 1}^{N}$$

$\Phi_{\theta } \left( \cdot \right)$ refers to 3D-ResNet and $\theta$ refers to network parameters.

In order to capture the action information $V_{ij}^{xyz}$ in multiple consecutive frames in boxing sports video, features are calculated from spatial and temporal dimensions. The value of the unit whose position coordinate is (*x, y, z*) in the *j*-th feature map of the *i*-th layer, as shown in Eq. (6):

6 $$V_{ij}^{xyz} = f\left( {b_{ij} + \sum\limits_{r} {\sum\limits_{l = 0}^{{l_{i} - 1}} {\sum\limits_{m = 0}^{{m_{i} - 1}} {\sum\limits_{n = 0}^{{n_{i} - 1}} {w_{ijr}^{lmn} v_{{\left( {i - 1} \right)r}}^{{\left( {x + l} \right)\left( {y + m} \right)\left( {z + n} \right)}} } } } } } \right)$$

$n_{i}$ refers to the time dimension of the 3D convolution kernel. $w_{ijr}^{lmn}$ refers to the weight value of the convolution kernel whose position (*l, m, n*) is connected with the *r* feature map, and $v$ refers to the action information of each video segment.

In this study, ReLU function is used as activation function. This function can make the parameters of the model sparse, thus reducing over-fitting. In addition, it can also reduce the calculation of the model. ReLU activation function definition is shown as Eq. (7):

7 $$f\left( x \right) = \max \left( {0,x} \right) = \left\{ {\begin{array}{*{20}c} 0 & {x \le 0} \\ x & {x > 0} \\ \end{array} } \right.$$

The calculation of maximum pooling in the model is shown in Eq. (8):

8 $$V_{x,y,z} = \mathop {\max }\limits_{{0 \le i \le s_{1} ,0 \le j \le s_{2} ,0 \le k \le s_{3} }} \left( {\mu_{x \times s + i,y \times t + j,z \times r + k} } \right)$$

$\mu$ refers to the three-dimensional input vector, *V* refers to the output after pooling operation, and *s, t,* and *r* refer to the sampling step size in the direction.

For a certain input feature $f\left( {v_{ti} } \right)$, firstly, two convolution layers are respectively used to map $f\left( {v_{ti} } \right)$ into a *K* vector and a *Q* vector, as shown in Eq. (9):

9 $$\left\{ {\begin{array}{*{20}c} {K_{ti} = W_{K} f\left( {v_{ti} } \right)} \\ {Q_{ti} = W_{Q} f\left( {v_{ti} } \right)} \\ \end{array} } \right.$$

$W_{K}$ and $W_{Q}$ are the weight matrices corresponding to the two convolution layers, the *Q* vector and the *K* vector of node $v_{ti}$, respectively. Next, the inner product of the sum of $Q_{ti}$ and $K_{ti}$ is calculated, as shown in Eq. (10):

10 $$u_{{\left( {t,i} \right) \to \left( {t,j} \right)}} = \left\langle {Q_{ti} ,K_{tj} } \right\rangle$$

Nodes $v_{ti}$ and $v_{tj}$ are in the same time step. $\left\langle , \right\rangle$ stands for inner product symbol. The inner product $u_{{\left( {t,i} \right) \to \left( {t,j} \right)}}$ is called the similarity between nodes $v_{ti}$ and $v_{tj}$.

In order to improve the generalization ability of the model, it is necessary to normalize the coordinate data involved in the calculation based on the image resolution, as shown in Eq. (11):

11 $$\left\{ {\begin{array}{*{20}c} {x^{\prime}_{i} = \frac{{x_{i} }}{{x_{width} }}} \\ {y^{\prime}_{i} = \frac{{y_{i} }}{{y_{height} }}} \\ {z^{\prime}_{i} = \frac{{z_{i} }}{{z_{lenght} }}} \\ \end{array} } \right.$$

$x_{i} ,y_{i} ,z_{i}$ are the abscissa, vertical coordinate and vertical coordinate of the key point *i* in the image. $x_{width}$, $y_{height}$ and $z_{lenght}$ are the width, height and length of a frame image. $x_{i}{\prime} ,y_{i}{\prime} ,z_{i}{\prime}$ are the normalized abscissa, vertical coordinate and vertical coordinate of the key point *i*.

**Figure 3:**
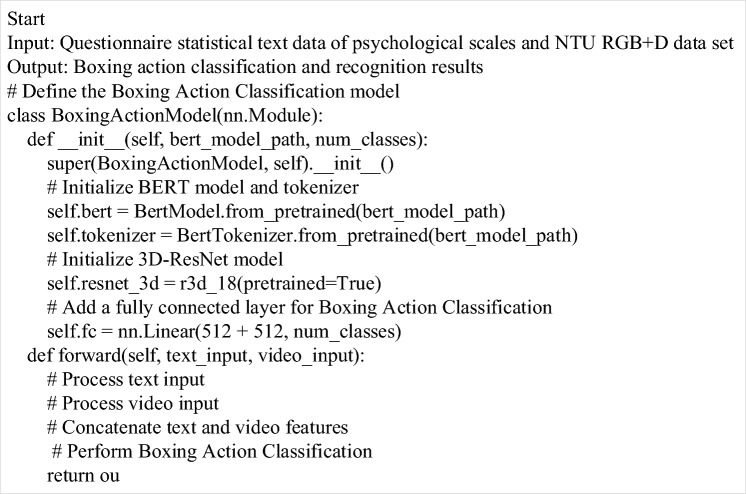
A pseudo-code flow chart of BERT fusion 3D-ResNet algorithm applied to boxing action classification and recognition.

Therefore, by learning the weights of any two body joints in different boxing movements, this data-driven way increases the universality of the model, so that the model can effectively identify and predict actions in the face of diverse data. The pseudo code of this model is shown in Fig. 3.

## Experimental design and performance evaluation

### Datasets collection

The data sources of this study include statistical text data of psychological scale and video data in NTU RGB + D dataset (NTU RGB + D dataset). The NTU RGB + D dataset contains 60 kinds of actions, including depth map, 3D bones joint position, RGB frame and infrared sequence. It is worth noting that the NTU RGB + D dataset itself does not directly contain the category labelled "Boxing Action". Therefore, an innovative method is adopted to screen and define action videos close to boxing action characteristics.

Specifically, the action categories in the dataset are comprehensively analyzed, and a series of actions that can represent or approach the basic boxing actions (such as hitting, defensive posture, etc.) are selected. Through further processing and combination of the selected actions, such as the splicing of action fragments and speed adjustment, a subset of actions that can simulate the characteristics of boxing actions is successfully constructed. In particular, the scope of the dataset has been expanded, including video data collected from actual boxing matches and training scenes. Through cooperation with professional boxing training centres, carefully selected video clips are obtained, which cover boxing techniques from basic to advanced, ensuring the diversity and richness of data. These additional collected data not only provide valuable samples for AI model training, but also increase the universality and accuracy of the research results.

Based on this process, 2169 bone samples are finally obtained, which are used to represent "boxing action" in this study. These text data and image data are divided into training set and test set according to data category, and the ratio is 8:2. The method not only allows to overcome the limitation of the original classification of datasets, but also provides an effective way for the identification and analysis of boxing movements by creatively reorganizing and utilizing existing data.

Although using text data alone to identify boxing behavior, text data (such as athletes' training logs, competition comments, etc.) can provide valuable information about boxing action types, strategies and competition situations. The advantage of this method is that it can capture the psychological and strategic details behind the action, which is difficult to achieve only by image data. However, its main limitation lies in the lack of direct visual evidence of the quality of action execution, which may lead to limited accuracy of action recognition. On the other hand, the method of combining text data and image data aims to make comprehensive use of their advantages. Image data (such as video frames) can provide intuitive visual information of action execution, which is helpful to capture the accuracy and technical details of action. The text data supplements the psychological and strategic insights behind the action. Therefore, the combination of the two methods can not only improve the accuracy of action recognition, but also increase the understanding of psychological motivation and strategy choice behind action execution.

### Experimental environment

To verify the algorithm, the hardware environment tested in this study is Intel i7-9750H CPU, 32 GB memory and NVIDIA RTX2070-8 GB GPU. Operating system: Windows 10 Professional Edition. Programming language: python. Development platform: pycharm. A platform for constructing convolutional neural network framework: pytorch.

### Parameters setting

The specific parameters are set as follows: the batch size is 100, with 80 iterations, and the loss function is optimized by random gradient descent algorithm, and the initial learning rate is set to 0.001. The optimizer is adma. In order to alleviate the phenomenon of over-fitting, the Dropout technique is used, and the Dropout ratio is set to 0.2, 0.4, 0.5, 0.6 and 0.8 for experiments.

### Performance evaluation

#### Statistical analysis of questionnaire survey results

**Figure 4:**
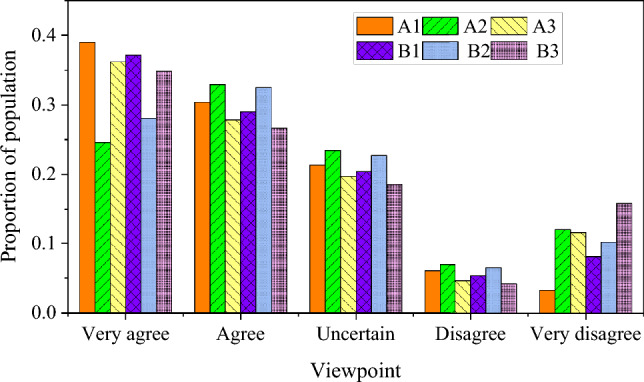
Survey results of anxiety level and self-confidence dimension.

**Figure 5:**
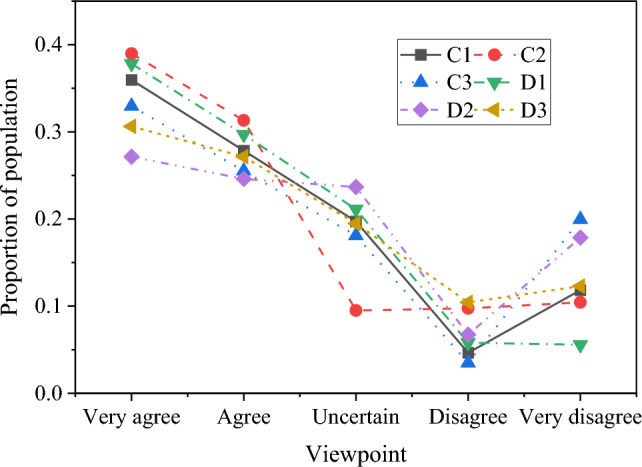
Survey results of team identity and opponent attitude dimensions.

The results of boxers' anxiety level, self-confidence, team identity and opponent's attitude obtained by psychological scale are statistically analyzed, as shown in Figs. 4 and 5.

In Figs. 4 and 5, the detailed results of four key psychological dimensions, namely, anxiety level, self-confidence, team identity and opponent's attitude, are obtained through the psychological scale survey of boxers. According to the data analysis of the scale, it is found that more than 55% of boxers feel high anxiety before the game or in the face of strong opponents, while 25% of boxers are relatively calm. In terms of self-confidence, more than 60% of athletes believe that they have enough skills and strength, but there are some differences between staying calm and self-confidence at critical moments, and 20% of athletes say that there may be some tension at critical moments. In terms of team identity, 72% athletes think that their team is a close cooperative group, and in difficult times, team support has a positive effect on athletes. In the aspect of opponent's attitude, 58% athletes show confidence in facing strong opponents, and at the same time think that the improvement of opponent's strength is an opportunity for personal progress.

#### Accuracy analysis of model classification and recognition under different algorithms

**Figure 6:**
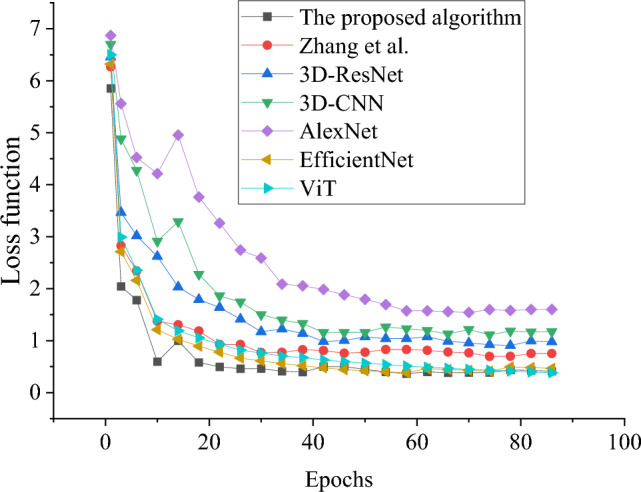
Results of model convergence performance under different algorithms.

**Figure 7:**
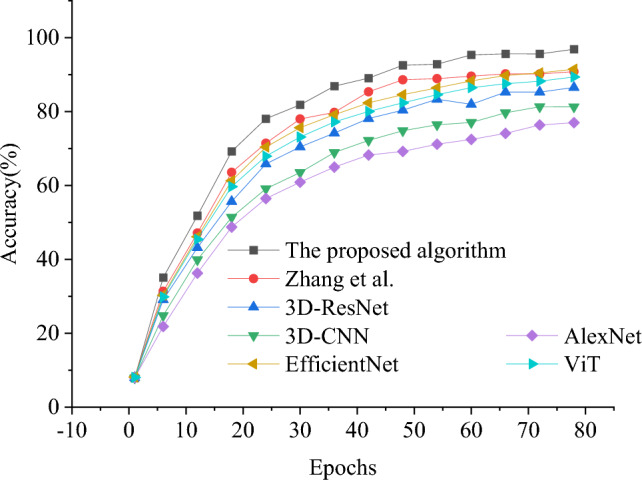
Accuracy result chart of boxing behavior classification recognition with iteration period under each algorithm.

**Figure 8:**
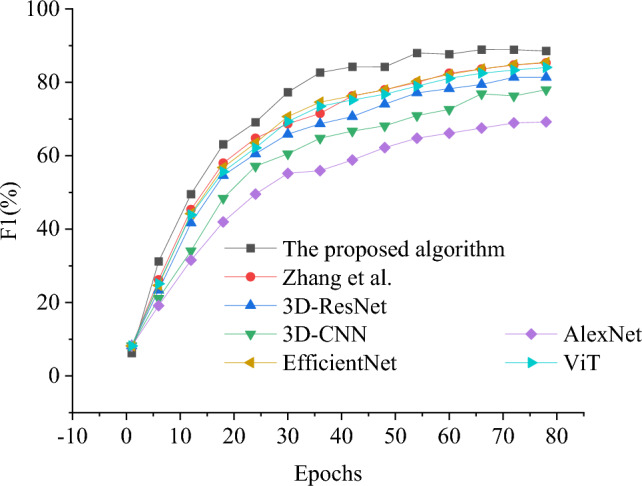
F1 value result chart of boxing behavior classification recognition with iteration period under each algorithm.

In order to evaluate the performance of the model in this study, the model algorithm in this study is compared with the model algorithm proposed by 3D-CNN^45^, 3D-ResNet, ResNet^46^ and Zhang et al.^32^, and evaluated from loss value, accuracy and F1 value respectively, as shown in Figs. 6, 7 and 8.

In Fig. 6, by analyzing the loss value of each algorithm, it shows that the algorithm used in this research model has the smallest loss value. In particular, the model algorithm in this study reaches a basically stable state when the iteration period is 22, and the loss value remains at about 0.49. However, the final loss functions of other algorithms, including the newly added EfficientNet and the visual model (ViT) based on Transformer, all exceed 0.75, among which the loss values of EfficientNet and ViT model are about 0.42 and 0.56 at the iteration period of 50, respectively. Compared with the boxing action classification and recognition model based on BERT fusion 3D-ResNet proposed in this study, the latter shows better convergence effect and its loss value is obviously lower. Therefore, even considering the latest EfficientNet and ViT models, the model proposed in this study still shows superior performance. This proves that the boxing action classification and recognition model based on BERT fusion 3D-ResNet has better accuracy and efficiency in dealing with complex action recognition tasks, which further strengthens the practical application value and scientific research significance of the model.

Further analysis of the classification accuracy of each algorithm, as shown in Figs. 7 and 8, with the increase of iteration period, the accuracy and F1 value of each algorithm first increase and then tend to be stable. The model algorithm in this study performs best in all comparisons, with an accuracy rate of 96.86%, which is at least 6.04% higher than other models, and the F1 value reaches 88.55%, which highlights its outstanding performance in boxing action classification and recognition. Although the newly introduced EfficientNet and Transformer-based ViT show potential in the task of action classification, this research model, with its unique structural design and effective integration of deep learning technology, not only keeps ahead in accuracy, but also shows obvious advantages in convergence speed and stability of the model.

In particular, this research model optimizes the process of feature extraction and action recognition by combining BERT and 3D-ResNet, so that the model can understand and deal with complex action patterns in time series data more effectively. This is especially obvious in the performance stability and accuracy improvement under high iteration period, which shows the superiority of this model in dealing with long-term dependence and action recognition. Therefore, the boxing action classification and recognition model based on BERT fusion 3D-ResNet is not only innovative in theory, but also shows excellent performance and broad application prospects in practical application.

#### The influence of psychological state on the recognition performance of boxing action

A series of ablation experiments are also designed to evaluate the contribution of each component of boxing action classification and recognition model based on BERT and 3D-ResNet fusion to the final performance. In this way, it aims to prove the importance of each component and show the innovation of the model. The model is mainly composed of two parts: BERT model is used to process time series data and capture subtle changes in actions. 3D-ResNet model is used to process video frames and extract spatial features. In order to deeply understand the contribution of these two parts to the model performance, the following ablation experiments are carried out. Complete model: A complete model combining BERT and 3D-ResNet. No BERT: Remove the BERT component and only use 3D-ResNet to process video data. No 3D-ResNet: Remove the 3D-ResNet component and only rely on BERT to process time series data.

Table 2 shows the accuracy and F1 value of the model on the test set under different ablation settings:

**Table 2:** Ablation experimental results.

Experimental setup	Accuracy rate (%)	F1 score (%)
Complete model	96.86	88.55
Only BERT	79.34	72.89
Only 3D-ResNet	85.76	78.45
BERT + simplified 3D-ResNet	93.27	85.67
Simplified BERT + 3D-ResNet	91.58	83.92

Table 2 shows that the complete model performs best in accuracy and F1 score, which further confirms the importance of the synergistic effect of BERT and 3D-ResNet components to improve the model performance. Compared with using 3D-ResNet alone, the performance of using BERT alone has declined, which may be because it is difficult for BERT to process complex video data independently when it lacks the spatial feature extraction support of 3D-ResNet. By simplifying BERT or 3D-ResNet, it is found that the performance of the model has declined, but it still maintains a high accuracy and F1 score. This shows that although the simplified components can still work effectively, the integrity of the model is necessary to achieve optimal performance. After simplification, BERT's contribution seems to have a greater impact on the performance of the model, which may be because BERT's ability to process time series data has a decisive impact on the overall performance.

#### The influence of psychological state on the recognition performance of boxing action

In order to deeply understand how psychological state affects the recognition performance of boxing movements, the accuracy of the model in dealing with boxing movements in different psychological states is comprehensively analyzed. Table 3 shows the key findings of the study, including the distribution of psychological state, the comparative analysis of model accuracy, and the specific influence of psychological state change on model recognition ability.

**Table 3:** Accuracy and time delay of model recognition under different mental states and boxing action types.

Psychology states	Boxing action type	Model recognition accuracy (%)	Average time delay (ms)
Low anxiety	Straight punch	94	100
	Hook	92	120
High anxiety	Straight punch	85	150
	Hook	80	170
High self-confidence	Straight punch	96	90
	Hook	95	110
Low self-confidence	Straight punch	88	130
	Hook	85	150
Strong team identity	Straight punch	93	95
	Hook	91	115
Weak team identity	Straight punch	87	140
	Hook	85	160
Positive opponent attitude	Straight punch	94	98
	Hook	92	118
Negative opponent attitude	Straight punch	86	148
	Hook	83	168

This table provides a more detailed perspective, showing how psychological state and boxing action type jointly affect the recognition accuracy and processing speed of the model. It shows that in the state of low anxiety and high self-confidence, the model can not only identify actions with higher accuracy, but also have a shorter average time delay, which indicates that a good psychological state may help athletes to perform more standardized and consistent actions, thus facilitating the rapid and accurate identification of the model. On the other hand, in the case of high anxiety or low self-confidence, not only the recognition accuracy decreases, but also the average time delay increases, which may be because the actions caused by these psychological states become more irregular or complex, which increases the difficulty of model processing. In addition, the data also shows the influence of team identity and opponent attitude on the performance of the model, which further confirms the important role of psychological factors in sports behavior analysis.

### Discussion

Through the psychological scale survey of boxers, it shows that detailed results have been obtained in anxiety level, self-confidence, team identity and opponent attitude, which provides practical data support for boxers' psychological training and psychological support, helps to formulate more personalized and effective training plans and improve athletes' psychological quality in the competition. This is consistent with Bozdarov et al.^47^ and Donnelly et al.^48^.

Meanwhile, the model algorithm proposed in this paper is compared with other model algorithms (uch as the model algorithm proposed by 3D-CNN, 3D-ResNet, ResNet and Zhang et al.^32^), and it shows that the accuracy of the model algorithm in this study reaches 96.86%, which is improved by at least 6.04%, and it is the best in accuracy. This is consistent with the views of Le et al.^49^ and Zhou et al.^50^. Therefore, by integrating the results of psychological scale, the model can understand the psychological state of athletes in the process of action execution more comprehensively, thus improving the classification accuracy of boxing actions. This provides a new method for the field of action recognition, and also provides useful practical experience for using deep learning technology to solve practical application problems.

### Ethical approval

The study was conducted in accordance with the Declaration of Helsinki, and approved by the Institutional Review Board of School of Science of physical culture and sports, Kunsan University (protocol code 2022.989432, approval date 2022.5.19).

### Consent to participate

Written Informed consent was obtained from all individual participants included in the study.

### Consent for publication

Written informed consent was obtained from the patient(s) for their anonymized information to be published in this article.

## Conclusion

### Research contribution

Through the psychological scale survey of boxers, this study deeply understands the psychological state of boxers such as anxiety level, self-confidence, team identity and opponent's attitude before the competition. By integrating the results of psychological scale, this paper constructs a boxing action classification and recognition model based on BERT fusion 3D-ResNet. It shows that the model not only performs well in loss value, but also outperforms other algorithms in accuracy, reaching more than 95%, which provides profound psychological background support for boxing action classification and recognition.

### Future works and research limitations

There are also some limitations in this study. Firstly, the results of the psychological scale are based on the subjective self-report of the questionnaire, which may have some subjective errors due to the subjective factors of the respondents. Secondly, this study only focuses on boxers, while athletes in other sports may have different psychological states and action characteristics. Therefore, the future research can further expand the sample size and cover more boxers with different levels and experiences to improve the representativeness and generalization ability of the research. Secondly, the method of this study can be extended to other sports fields to carry out more comprehensive research on sports psychology and action classification.

## Data Availability

The datasets used and/or analysed during the current study available from the corresponding author Yuanhui Kong on reasonable request via e-mail YuanHuiKong1996@163.com.
